# Neutrophils Culture in Collagen Gel System

**DOI:** 10.3389/fimmu.2022.816037

**Published:** 2022-01-24

**Authors:** Ru Li, Ziqing Wang, Junhao Huang, Sixiao He, Yanmei Peng, Yong Wan, Qiang Ma

**Affiliations:** ^1^ Department of Biopharmaceutics, School of Laboratory Medicine and Biotechnology, Southern Medical University, Guangzhou, China; ^2^ Department of General Surgery, Nanfang Hospital, Southern Medical University, Guangzhou, China; ^3^ Research and Development Department, Guangzhou Darui Biotechnology Co., Ltd, Guangzhou, China

**Keywords:** neutrophils, 3D culture system, hydrogel, NETs, co-culture

## Abstract

Neutrophils (Neu) migrate rapidly to damaged tissue and play critical roles in host defense and tissue homeostasis, including the intestinal epithelia injuries and immune responses. Although their important roles in these diseases, they are challenging to study due to their short life span and the inability to cryopreserve or expand them *in vitro*. Moreover, the standard cell culturing on plastic plates (two-dimensional (2D) cultures) does not represent the actual microenvironment where cells reside in tissues. In this study, we developed a new three-dimensional (3D) culture system for human and mouse peripheral blood Neu, which is made of hydrogel. The Neu showed much better cell integrity and less cell debris in the 3D culture system compared to that in 2D culture system. Moreover, the 3D culture system was more suitable for the observation of neutrophil extracellular traps (NETs) stimulated by the classical stimulation phorbol ester (PMA), and other damage associated molecular patterns (DAMPs) such as Lipopolysaccharide (LPS)/ATP, interleukin-1 β (IL-1β) and tumor necrosis factor α (TNFα) than the 2D culture system. Moreover, NETs phenomenon in 3D culture system is similar to that *in vivo*. In addition, the 3D culture system was evaluated to co-culturing Neu and other parenchymal cells, such as colon mucosal epithelial cell lines. In conclusion, the 3D culture system could maintain better properties of Neu than that in 2D culture system and it may reduce the gap between *in vitro* an *in vivo* experimentations.

## Introduction

Neu play crucial roles in elimination of pathogens and damaged tissue and cells, Neu depletion exacerbated colitis in the intestinal epithelia injury mode, and it plays a critical role in regulating the intestinal immune responses (1, 2). Once the Neu enters the tissue, it kills the bacteria through degranulation, phagocytosis, or extracellular traps (3, 4) that release DNA strands and bactericidal proteins. Although their important roles in these diseases, Neu have a relatively short lifespan and they will undergo apoptosis within 24 hours *in vivo* (5, 6). Moreover, the research on Neu is mainly based on fresh isolation (3), but they can only be kept alive within a short time frame *in vitro* (7). So prolonging the survival time of Neu and maintaining their activity *in vitro* is thus an important and urgent challenge (8).

The classical 2D monolayer cell culturing on flat and rigid substrate is a prevalent model system used to study immunological processes *in vitro*. But the 2D system was challenged in recent years by findings from the studies which revealed that the 2D-cultured cells are unable to simulate the exact phenomena observed *in vivo*, including topography, mechanics, and hierarchical tissue assembly, possibly resulting in non-physiological cell behavior (9–12). The drawbacks of the 2D system are also related to the investigations of inflammatory processes. During inflammation, Neu infiltrate tissues where they perform their effector functions. The tissues are three-dimensional and thus performance of the cells might also be different than in the 2D conditions (13). Some scholars have studied the 3D culture of neutrophil-like cell line HL-60 or primary Neu co-culture with other cells in collagen gels for improve the viability of Neu (12, 14–16), however, the detailed phenomena of Neu in 3D culturing system have never evaluated.

Hydrogels are composed of cross-linked hydrophilic polymer chains and can be used to form a scaffold for the construction of three-dimensional cell culture models. They are in a liquid state at 4°C and solidify at 37°C. They have good permeability, allowing a variety of nutrients, gases, and metabolites to pass through them freely and resembling the extracellular environment of the body’s tissues (17). The exiting studies have showed that the hydrogels were used for hepatocytes, cardiac cells, breast cancer cells, islet beta-cells, and colon cancer cells culturing (15, 18–22), and for organoid culture, providing models for disease research (23–26), however, the 3D culture system has never been used to cultivate Neu.

In this study, we have established their 3D culturing system with hydrogels and evaluated the detailed phenomena of Neu. We cultured human and mouse derived peripheral blood Neu in the 3D hydrogel, demonstrating better morphological activity, oxidation, more chemotaxis, higher activity and more generation of NETs with incubation in PMA, LPS/ATP, IL-1β and TNFα than those in 2D culture system. Furthermore, when stimulated by PMA, Neu exhibited still better NETs characteristics, similar to those seen in animal experiments. When co-cultured with colon mucosal epithelial cell lines, Neu could still maintain good characteristics, which provides a model for studying the inflammation of skeletal muscle innate immunity. Overall, the results suggest that this 3D system is suitable for culturing Neu, hoping to provide convenient simulation for *in vitro* studies on Neu.

## Materials and Methods

### Reagents

Adult male C57BL/6 mice, 8–10 weeks of age, were purchased from the Guangdong Medical Laboratory Animal Center (Guangzhou, China). The animals were housed in the animal center of the Southern Medical University, according to the criteria outlined in the Institutional Animal Care and Utilization Committee (IACUC). The mice (4 per cage) were housed under controlled environmental conditions (12 h light/dark cycle, 55% ± 5% humidity, 23°C ambient temperature) with free access to standard chow and water. All experimental protocols involving animals were approved by the Animal Care and Use Committee of the Southern Medical University in accordance with the Guide for the Care and Use of Laboratory Animals of the National Institutes of Health.

The hydrogel we got was Nitta Gelatin’s Type I collagen, and the concentrated medium was composed of Ham’s F-12 medium, MEM with Hanks’ balanced salt solution DF medium (DME: F-12 = 1: 1) and Medium 199, reconstitution buffer was composed of sodium hydroxide (50 mM), sodium bicarbonate (260 mM) and HEPES (200 mM). I-PC and I-AC collagen were purchased from KOKEN. Collagen type I was purchased from Sigma. Cells disperse enzymes from Kit for Cell Premedium which were gifts from DARUI BIO (Guangzhou, China). EGTA (Cat#324626) was purchased from Merk. PMA was purchased from Sigma-Aldrich. MTT assay kit (KGA312)was purchased from Keygenbiotech. The apoptosis kit (Cat#70-AP101) was purchased from MultiSciences. Z-VAD-FMK (V116) was purchased from Sigma and WKYMVm (ab141811), LY6G-APC-750 antibody (ab46754) and NE (ab68672) were purchased from Abcam. Quant-iT™ PicoGreen™ dsDNA Reagent, SYTOX^®^ green nucleic acid stain (S7020), SYTO^®^ 13 (S7575) and CD11b-FITC antibody (53-0112-82) were purchased from Invitrogen. Tubulin beta antibody (AF7011) was purchased from Affinity Biosciences. DNA ladder detection kit (KGA112) was purchased from Keygenbiotech. Dextran T-500 (D8270) was purchased from Solarbio. SuperRed/GelRed (BS354B) was purchased from Biosharp Life Science and DNA marker was purchased from Dongsheng Biotech.

### Murine Bone Marrow Neu Isolation

Neu were obtained from mouse bone marrow according to standard techniques. Briefly, mice were sacrificed by cervical dissection, the femur and tibia of the mice were taken, and the end of the bone was excised and the medullary cavity exposed, followed by the bone being washed with phosphate-buffered saline (PBS) with an 1 mL syringe and a 27-gauge needle, three times per bone. The washed suspension was filtered through a 100 µm filter, centrifuged at 400 xg for 10 min, and resuspended in 1 mL PBS. A Percoll stock solution (9 mL Percoll, 1 mL 9% NaCl) was diluted with 0.9% NaCl to give 54%, 64%, and 72% Percoll working solutions. After centrifugation (1000 xg, 30 min), the interface between the 64% and 72% layers containing Neu was harvested, with purity more than 95%.

### Mouse Peripheral Blood Neu Isolation

Mouse peripheral blood Neu were isolated from peripheral blood by density gradient centrifugation (Beijing Solarbio, P9201). Briefly, heparinized blood was sedimented with 6% dextran T500 in PBS for 30 min at 37°C, the top clear layer containing leukocytes was transferred to a fresh tube and the cells were underlaid with 2 mL of Agent C in the middle and Agent A down here, and centrifuged at 1000 xg for 30 min. Neu between Agent A and C were obtained, resulting in cell populations containing at least 90% Neu.

### Human Peripheral Blood Neu Isolation

Human blood was taken from healthy individuals following informed and written consent in a qualified institution. Human Neu were isolated from peripheral blood by density gradient centrifugation. Briefly, heparinized blood was sedimented with 6% dextran T500 in PBS for 30 min at 37°C, the top clear layer containing leukocytes was transferred to a fresh tube and the cells were underlaid with 10 mL of Ficoll Paque Plus, and centrifuged at 750 xg for 20 min. The Neu and erythrocytes were treated erythrocyte lysis solution (155 mM NH_4_Cl, 10 mM KHCO_3_, 0.1 mM EDTA, pH 7.3), resulting in cell populations containing at least 95% Neu.

### Gel Transparency and Adhesion Evaluation

To prepare a hydrogel for the 3D culture system, 0.1 mL of concentrated medium was added to the cooled 0.8 mL of Cellmatrix type I collagen which was at pH 3 with a concentration of 3 mg/mL on ice, mixing well to become light yellow, then additional 0.1 mL of reconstitution buffer was mixed well to become light pink. Last, the mixture was added to the six-well plate (100 μL/well), which was then was placed in a 37°C incubator for 45 min, followed by culture at 37°C in 2 mL of complete medium containing 10% FBS. For comparison, I-PC collagen and I-AC collagen from KOKEN and Collagen type I from Sigma were executed in the same way. In order to evaluate the transparency of the gel, we observed and compared the transparency of the four collagen. In order to evaluate the adhesion of the gel, we placed the six-well plate on a horizontal shaker and oscillated for 1 hour at a rotation speed of 100 rpm/min and observe whether the glue drops in the wells of the plate fell off.

### Cell Culture

To prepare a hydrogel for the 3D culture system, human Normal-Derived Colon Mucosa (NCM) Cell Lines NCM460 or mouse skeletal muscle cells C2C12 (2.5 × 10^6^/mL each) together with corresponding species Neu or Neu alone were mixed in 0.8 mL hydrogel, 0.1 mL buffer, and 0.1 mL 10 x fetal bovine serum (FBS) on ice. The gel was placed in a six-well plate (100 μL/well), which was then was placed in a 37°C incubator for 45 min, followed by culture at 37°C in complete medium containing 10% FBS. For the 2D culture system, Neu together with NCM460 or C2C12 were cultured according to traditional culture methods.

If digestion needed, the hydrogel was washed once with pre-chilled neutral PBS and incubated with cell dispersase (diluted 10-fold with basic medium) at 37°C incubator for about 30 min until the hydrogel was completely digested. Finally, the digestion was stopped with the same amount of complete medium. After remove the supernatant at 250 xg for 5 min centrifugation, we stopped digestion again with 5 mL of 0.38 mg/mL EGTA and centrifuge at 250 xg for 5 min to obtain cell pellets.

### MTT Assay

The MTT colorimetric method was used to measure cell viability to quantify cells in small droplets of collagen. According to the standard operation in the manual, 100 μL per well (about 1 × 10^5^) of cells for 2D culture and 3D culture (2 μL of collagen) were added to a 96-well plate, and incubated in a 37°C with 5% CO_2_ cell incubator for 24 h. The Neu cultured in hydrogel system were digested and then suspended in 100 μL medium. Then 5 × MTT was diluted to 1 × MTT by Dilution Buffer. Followed by adding 50 µL 1 × MTT to each well and incubating at 37°C for 4 h to reduce MTT to formazan. After the supernatant was aspirated, 150 µL of DMSO were added to each well to dissolve formazan, and shaked well with a plate shaker. The microplate reader detected the optical density of each well at a wavelength of 490 nm.

### Analysis of Cell Activity and Nuclear Morphology

Human or mouse peripheral blood Neu (5 × 10^6/^mL) were cultured for 6 days in 2D and 3D culture systems with or without the inhibition of apoptosis z-vad-fmk (10 μM), stained with PI at room temperature in the dark for 15 min, and stained then with SYTO 13 for 15 min. The cells were washed twice with PBS, examined and immediately photographed under a fluorescence microscope. Dead cells were defined on the basis of positive PI staining and nuclear morphology changes such as chromatin condensation and fragmentation.

### Isolation of Genomic DNA and Gel Electrophoresis

Genomic DNA was isolated from mouse Neu according to the manufacturer’s protocol (DNA ladder detection kit, Keygenbiotech). Briefly, 5 × 10^5^ cells collected by centrifugation at 280 xg for 5min were treated with 50 μL lytic buffer and mixed vigorously, followed by added 5 μL RNase A (37°C, 30 min) and proteinase K (50°C,1 h). DNA in the aqueous phase was precipitated at -20°C in 40 μL sodium acetate and 200 μL 100% ethanol. Precipitates were pelleted by centrifugation (12,000 xg, 10 min, 4°C), washed with ice-cold 70% ethanol, and dried.

For electrophoresis, DNA samples were dissolved in 10 μL of Tris-EDTA buffer. Gel loading buffer was added, and the samples were subjected to electrophoresis in 2% agarose gel containing SuperRed/GelRed (1:10000) at 4 V/cm for 2 h. DNA was visualized by UV light and photographed. The analyzed DNA fragments in the samples were compared with standard size fragments of the DNA marker LM1061.

### ROS Measurements

For the detection of intracellular ROS level, ROS-sensitive probe H2DCFDA was used. Neu obtained from 2D culture system by direct centrifugation or 3D culture system by digestion (as shown in Cell Culture part) were incubated with 5 µM staining solution in PBS in the dark for 30 min at 37°C. Cells were centrifuged to remove supernatant, suspended in a fresh medium and immediately analyzed with flow cytometer (CytoFLEX, BECKMAN).

### Detection of Chemotaxis

Migration assays were conducted using a 24-well Transwell chamber (Corning, New York, US) with 4 μm pores. One × 10^6^ Neu cultured for 3 days in 2D and 3D culture systems were loaded (200 μL of a 5 × 10^6^/mL solution) into the top chamber, with 300 μL WKYMVm (100 nM) for mouse peripheral blood Neu, fMLF (1 μM) for human peripheral blood Neu or DMEM contained 1% bovine serum albumin, as control, in the lower chamber, and then the assay system was incubated for 3 h at 37°C. Migrating cells dropped from the filters to the bottom of the lower chamber. After 350 xg for 10 min centrifugation, the cells on the chamber were counted in five fields (magnification × 200) using a light microscopy equipped with a image analyser.

### Detection of NETs

Neu cultured in 2D and 3D systems for 3 days were stimulated with 20 μM PMA (1:810) for 4 h and then fixed with 4% paraformaldehyde for 15 min, permeabilized with 0.25% TritonX-100 for 10 min, blocked with PBS containing 5% bovine serum albumin for 1h, stained with NE (1:100), Sytox green (1:5000), DAPI (1:100), or SYTO 13 (1:5000) before being examined under a confocal or fluorescence microscope. To observe the production of NETs in live LPS-induced mice, we labeled Neu with LY6G-APC-750 (10 μg) and the DNA of NETs with Sytox green (10 nmol) *via* tail vein injection in advance, and NETs were observed under a two-photon fluorescence microscope (FV1200MPE, Olympus).

In order to determine the content of the above-mentioned NETs, dsDNA in the supernatants was evaluated by adding 100 μL Quant-iTTM PicoGreen^®^dsDNA Reagent (Invitrogen, 1:200 in 1× TE) to 100 μL sample as recommended by the manufacturer, followed by immediate measurement of fluorescence at 485 nm excitation, 520 nm emission with fluorescence plate reader FluoStar.

For the measurement of neutrophil elastase coupled dsDNA release, NE antibody (100 ng per well) was coated on the 96-well plate overnight, then 100 μL sample and 100 μL Quant-iTTM PicoGreen^®^dsDNA Reagent (Invitrogen, 1:200 in 1 × TE) were added as recommended by the manufacturer, followed by immediate measurement of fluorescence at 485 nm excitation, 520 nm emission with fluorescence plate reader FluoStar.

### Statistical Analysis

Neu apoptosis and functional results are given as mean ± SD. Differences were analyzed by the Two-way ANOVA with Tukey post-test using Prism version 7 (GraphPad). A *p*-value of 0.05 was considered to indicate a significant difference.

## Results

### Evaluation of Properties of the Cellmatrix Type I

**Figure 1:**
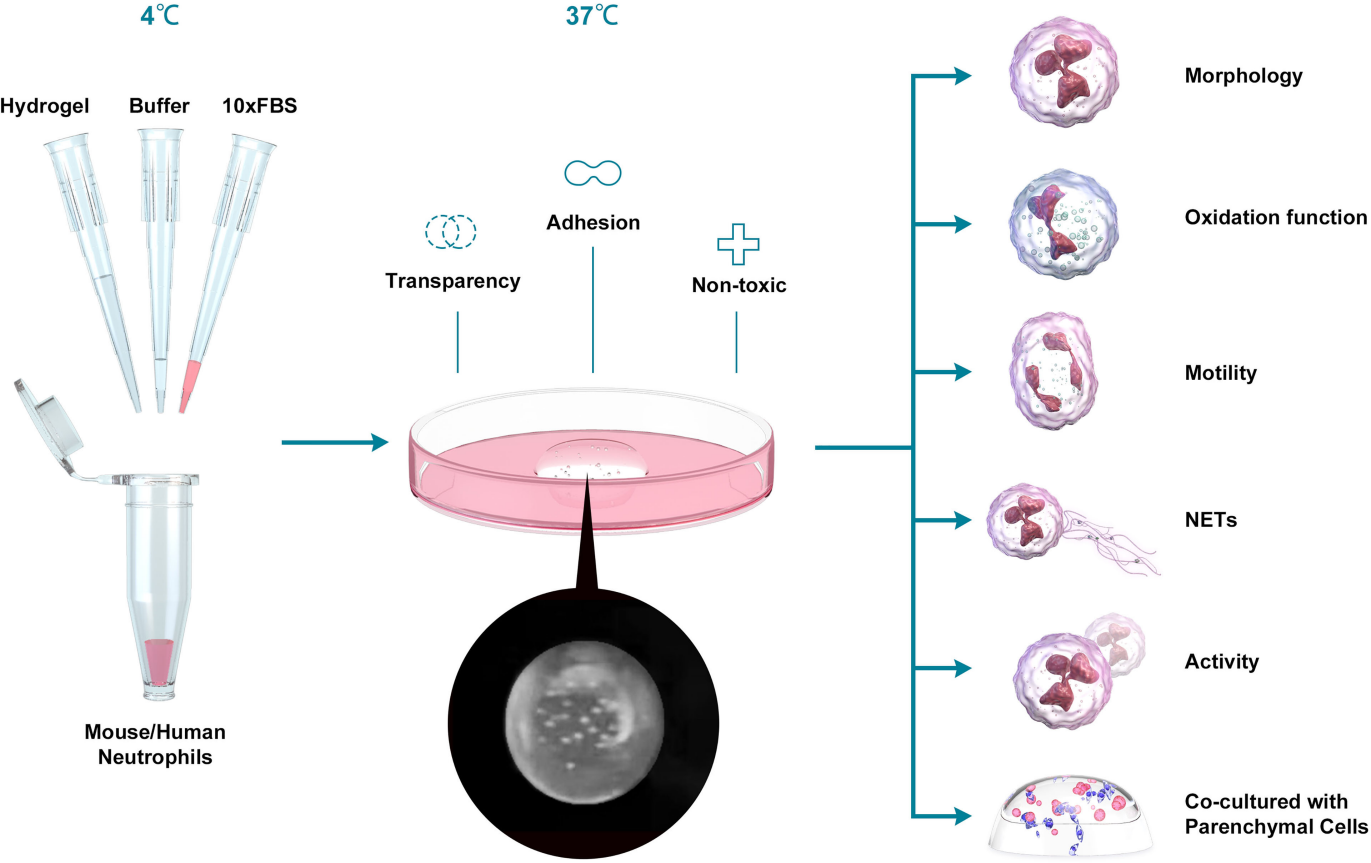
Schematic depiction of the experiment. We mixed hydrogel, buffer, FBS and Neu, incubated at 37 °C for 30 min and added the medium to culture the cells. Then we tested the oxidation function, the motility, the NETs production, activity and the ability to co-culture with Parenchymal cells between Neu in 2D and 3D culture systems.

**Figure 2:**
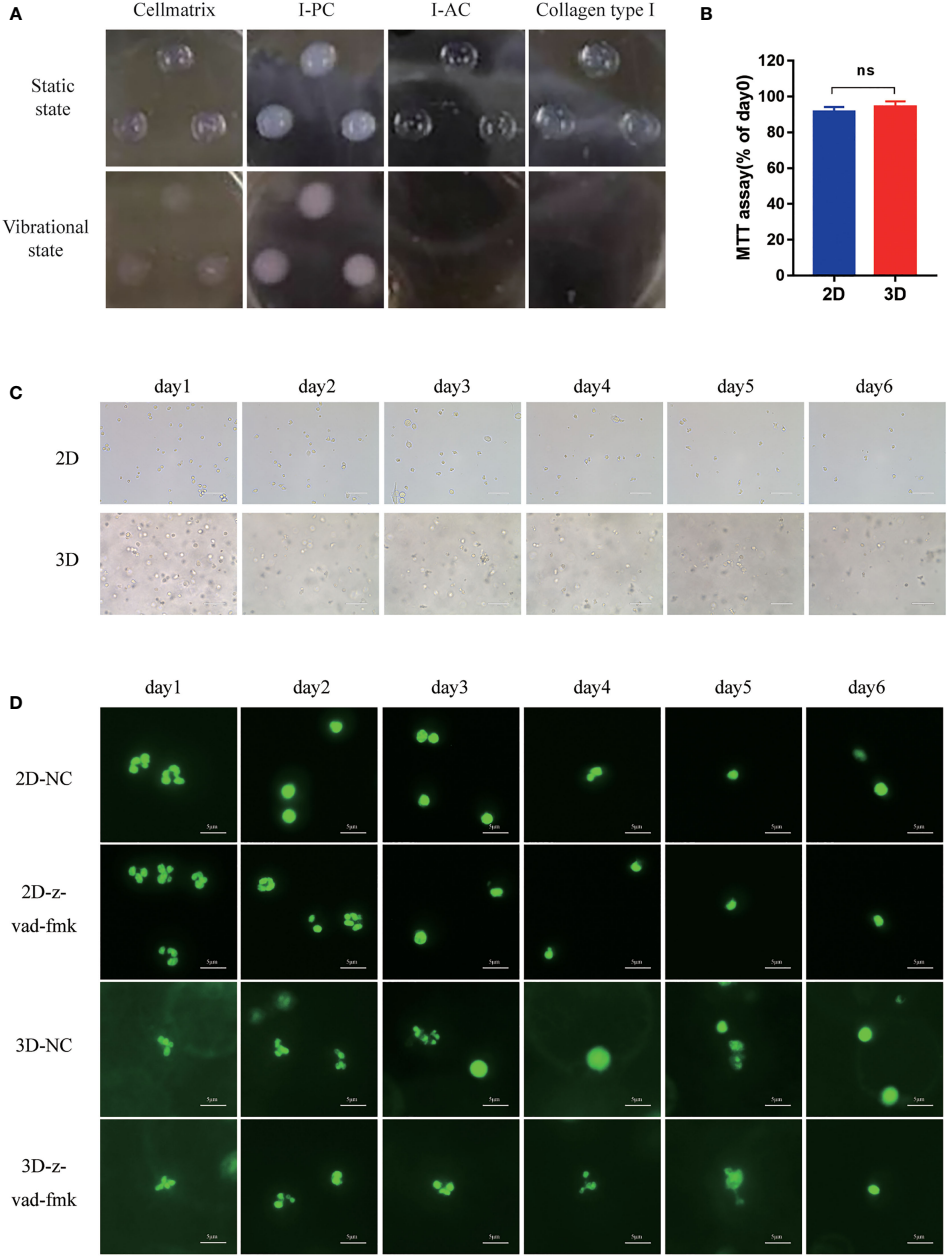
The morphology of Neu in 2D and 3D culture systems. **(A)** Morphology of Cellmatrix Type I, I-PC collagen and I-AC and Collagen type I in a static state or vibrational under light microscope. **(B)** Toxicity testing was evaluated by the MTT assay. Mouse bone marrow-derived Neu were embedded in Cellmatrix Type I and cultured for 24 h. MTT reagent was used to detect the survival rate of the cells. **(C)** The morphology of mouse peripheral blood Neu representative light microscopy photographs (600x). **(D)** Representative fluorescence micrograph of human peripheral blood Neu dyed by SYTO 13 (stain DNA in both live and dead eukaryotic cells) during 6 days in 2D and 3D culture systems with or without the inhibition of apoptosis z-vad-fmk. Error bars represent mean ± S.D. n = 3, ns, not statistically significantly different by One-way ANOVA.

As shown in **Figure 1**, we evaluated the performance of the gel and the Neu embedded in it. Cellmatrix type I collagen has been used to culture mesenchymal stem cells by other researchers before (27). We examined the characteristics of the four collagen gels before exploring the role of collagen gels in the cultivation of Neu. As shown in **Figure 2A**, the shape of the gel droplets were full and round hemispheres, obviously, Cellmatrix type I, I-AC and collagen type I showed better transparency, but I-PC did not, I-AC. Collagen type I was easy to fall off after shock, and the adhesion was poor. So we chose Cellmatrix type I for Neu culture to ensure we could observe the hydrogel with microscope.

The cytotoxicity of Cellmatrix type I collagen was evaluated by MTT assay to compare Neu activity after cultured in 2D culture medium or in 3D hydrogel with the same culture medium for 24 h. As shown in **Figure 2B**, there was no significant difference in Neu activity in 2D and 3D cultures after 24 h.

### Morphology of the Fresh Neu in the 3D Culture System

Mouse peripheral blood Neu had round shape and were in a full state during the first day, but developed incomplete shapes with large amounts of cell debris on the second day in the 2D system. By contrast, cells remained intact in the 3D system (**Figure 2C**).

In order to evaluate the cell status more intuitively, we stained the human Neu nucleus with SYTO 13 which stained DNA in both live and dead eukaryotic cells and observed it under a fluorescence microscope (**Figure 2D**). Neu in all groups maintained intact multilobed nuclear structure on the first day, but nuclear condensation and loss of chromatin appeared on day2 in 2D culture system, while this phenomenon was not observed in the group with the addition of apoptosis inhibitor z-vad-fmk. Neu still had intact multilobed nuclear structure until day 4 in 3D culture system, which suggested that 3D culture may protect Neu from apoptosis.

### Function of the Fresh Neu in the 3D Culture System

**Figure 3:**
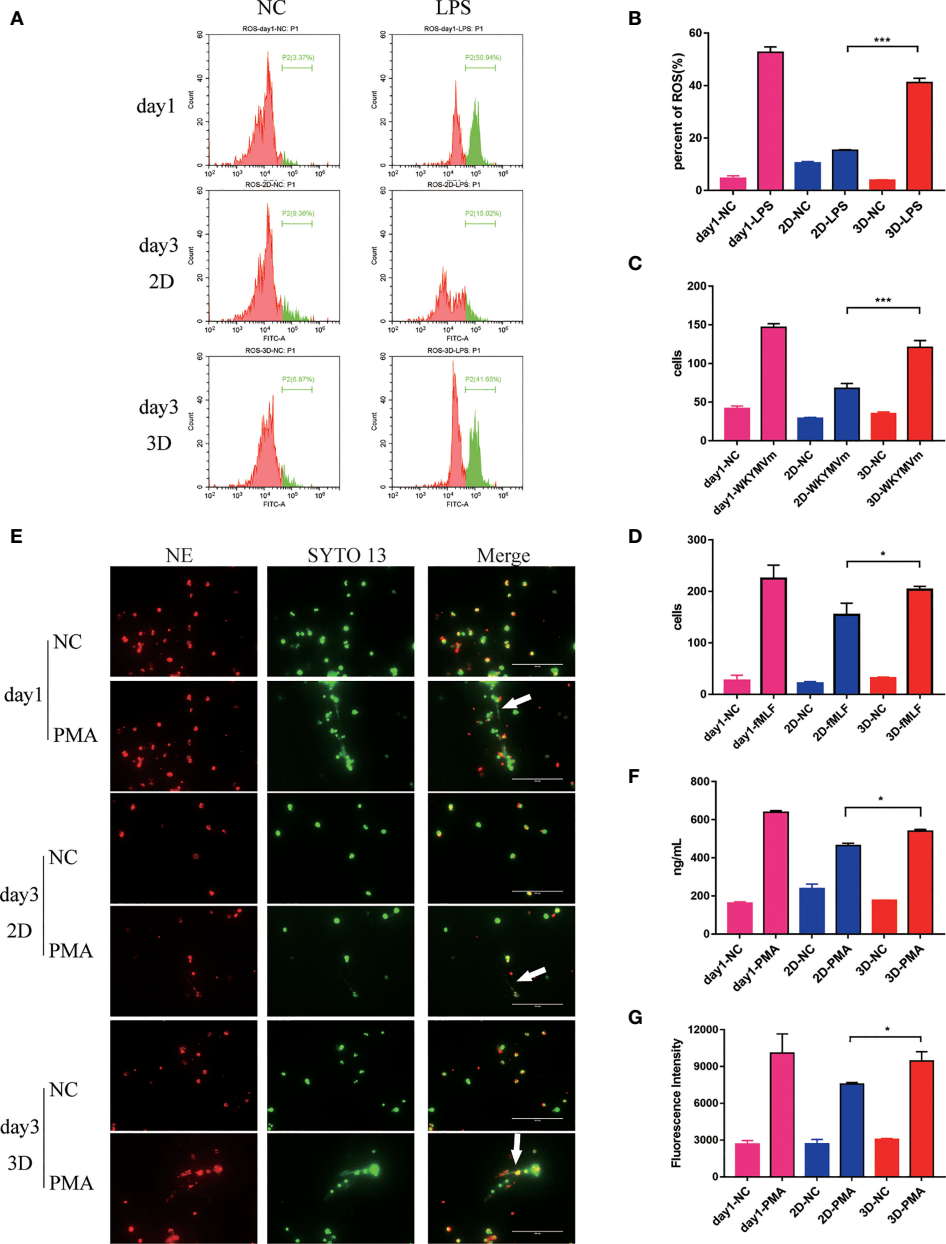
The evaluation of function of Neu during 6 days in 2D and 3D culture system. **(A)** Flow cytometry analysis of ROS produced by mouse peripheral blood Neu cultured in 2D and 3D systems and corresponding statistical results **(B)**. Chemotactic ability of mouse peripheral blood Neu shown as the number of cells per high power field on the Petri dish bottom in 2D culture and 3D culture systems for control (randomly migrating) and WKYMVm (100 nM) stimulated mouse peripheral blood Neu **(C)** or control and fMLF (1 μM) stimulated human peripheral blood Neu **(D)**. **(E)** Human Neu released NETs marked by NE (Alexa Fluor 594 dye, red) and nuclear (SYTO 13, green) when stimulated by 20 μM PMA for 4 h in 2D and 3D culture systems under a confocal microscope *in vitro*. **(F)** A significantly different dsDNA release was detected using fluorescent-based Picogreen assay. **(G)** Meanwhile, a significantly different release of neutrophil elastase coupled dsDNA was measured using fluorescent-based Picogreen assay coated with NE antibodies. Error bars represent mean ± S.D. n = 3, *p < 0.05, **p < 0.01, ***p < 0.001, ****p < 0.0001, ns, not statistically significantly different by Two way ANOVA.

The intracellular ROS level was detected by the fluorescent probe H2DCFDA. As shown in **Figures 3A, B**, under the same LPS stimulation, 3D cultured mouse peripheral blood Neu produced more ROS than that in 2D culture system, although lower than the first day.

To evaluated the chemotaxis of Neu in 2D and 3D culture systems, we conducted the migration assays. As expected, Neu in 3D culture system migrated more to the lower chamber stimulated by WKYMVm (100 nM) for mouse peripheral blood Neu and fMLF (1 μM) for human peripheral blood Neu for 3 h than that in 2D culture (**Figures 3C, D**).

In addition, Neu capture microorganisms, activate myeloid cells, and promote coagulation through NETs, which contain chromatin decorated with specific proteins (28, 29). Studies have shown that Neu produced NETs when stimulated by PMA (30). As shown in **Figure 3E**, human peripheral blood Neu released large amounts of dsDNA and NE when stimulated by 20 μM PMA for 4 h. Surprisingly, we found that more NETs were produced (white arrow) in 3D than that in 2D culture, a similar phenomenon in mouse peripheral blood Neu (**Supplementary Figure 1A**). To quantify dsDNA with a general method (31), we measured the fluorescence of supernatants which were from PMA stimulated mouse Neu binding with PicoGreen^®^dsDNA Reagent. As for the NE-coupled dsDNA, we coated NE antibody (100 ng per well) on the 96-well plate overnight in advance. Obviously, Neu in 3D culture system released more dsDNA and NE-coupled dsDNA, which were significantly different (**Figures 3F, G**).

A significant increase of NETs were seen following stimulation of mouse peripheral blood Neu with LPS/ATP, IL-1β and TNFα (**Supplementary Figures 1B, C**). Similarly, 3D cultured Neu produced more NETs than 2D cultures.

### Activity of the Fresh Neu in the 3D Culture System

**Figure 4:**
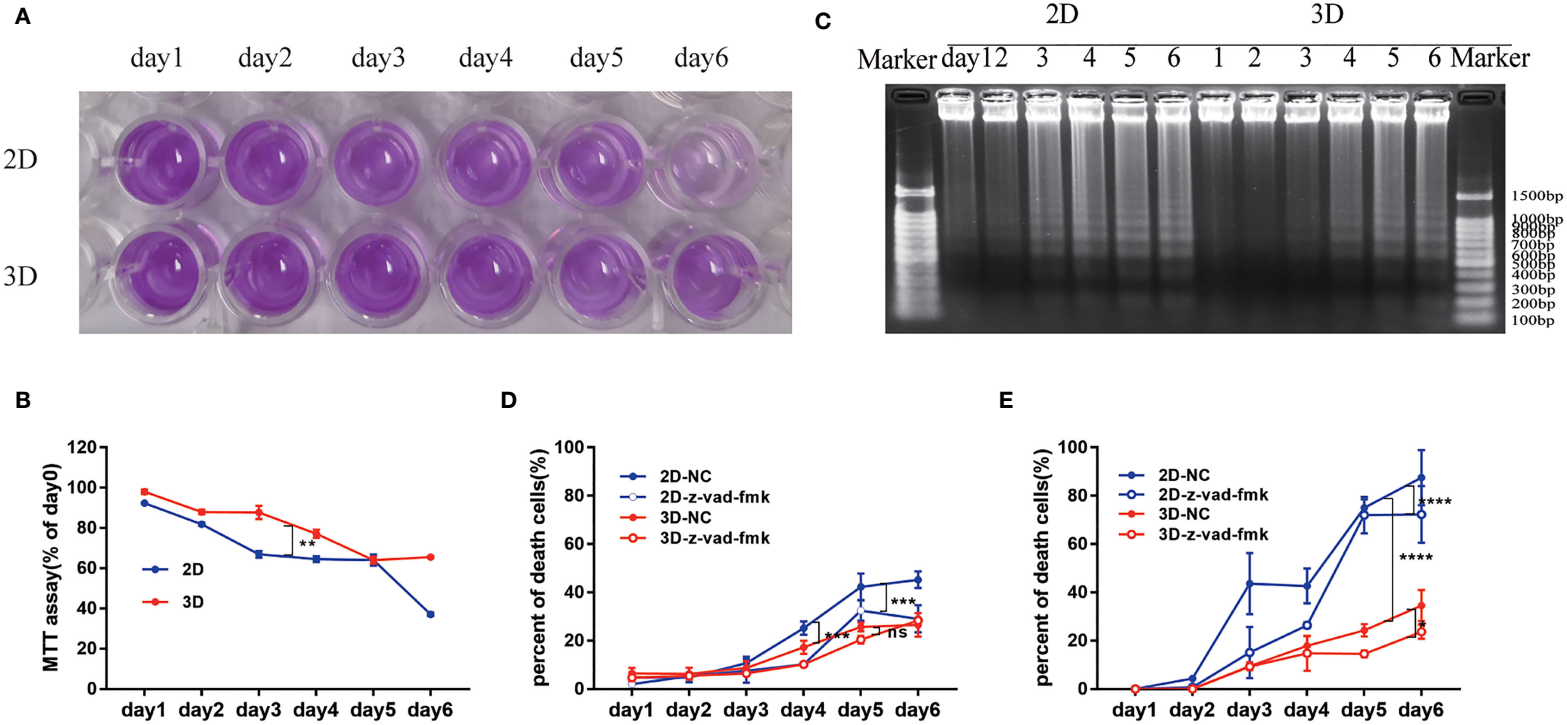
The activity of Neu in 2D and 3D culture systems. **(A)** Survival rate was evaluated by the MTT assay. **(B)** Percentage of the absorbance value relative to the cells on day 1 was used to indicate the active cells. **(C)** DNA integrity was determined by electrophoresis of DNA isolated from cultured mouse Neu for 1-6 days. Each slot was loaded with 50 μg of genomic DNA (corresponding to 5x10^5^ Neu used for preparation). Percentage of PI positive in the total mouse **(D)** and human **(E)** peripheral blood Neu which were dyed by SYTO 13 during 6 days in 2D and 3D culture systems with or without the inhibition of apoptosis z-vad-fmk was used to indicate the dead cells. Data from one of three experiments performed are shown. Error bars represent mean ± S.D. n = 3, *p < 0.05, **p < 0.01, ***p < 0.001, ****p < 0.0001, ns, not statistically significantly different by Two way ANOVA.

The activity of mouse bone marrow-derived Neu evaluated by MTT assay in the 2D culture system decreased rapidly, which was significantly slower than that of the 3D culture system (**Figures 4A, B**).

It is well known that caspase or other proteins activated early in apoptosis will activate degrading enzymes, which begin to cut DNA in the linker region, forming nucleosome and oligonucleosome DNA fragments (180 bp and multiples of 180 bp) (32). To assess the degree of Neu apoptosis during culture in 2D and 3D systems, we detected the classic approach of DNA fragmentation of 180 bp and multiples thereof (33). The characteristic “DNA ladder” pattern can be observed in **Figure 4C**. And apoptosis occurred on the 2nd day in 2D culture system, but on the 4th day in 3D culture system, which could be consistent with the appearance of nuclear condensation in **Figure 2D**.

We also detected cell viability by observing the co-localization of SYTO 13 (stain DNA in both live and dead eukaryotic cells) and PI (stain DNA in dead eukaryotic cells) under a fluorescence microscope (**Supplementary Figures 2**, **3**), and the ratio of PI to SYTO 13 was used to evaluate the percentage of dead cells. As shown in **Figures 4D, E**, the Neu of mouse or human in 2D culture system behaved more apoptosis than that in 3D culture system taken for comparison on the same day. The inhibition of apoptosis z-vad-fmk could easily alleviate the death of cells in 2D culture systems (***p<0.001). Interestingly, the cell viability of human peripheral blood Neu was higher than that of mice with the same culture time and culture system (**Figures 4D, E**).

### Status of the Fresh Neu When Co-Cultured With Parenchymal Cells in 3D Culture System

**Figure 5:**
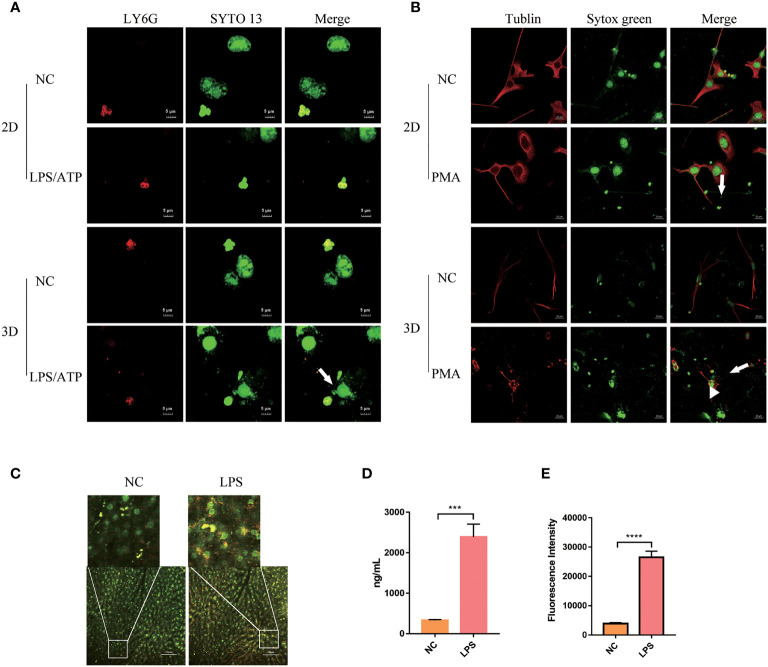
Peripheral blood Neu co-cultured with parenchymal cells in 2D and 3D culture systems. **(A)** The formation of NETs (white arrow) of human peripheral blood Neu co-culture with NCM460 were observed under a confocal microscope. LY6G, PE (red) and nuclear, SYTO 13 (green). **(B)** The formation of NETs (white arrow) of Neu and blebbing (white arrowhead) of C2C12 were observed under a confocal microscope. Tublin, Alexa Fluor 594 dye (red) and nuclear, sytox green (green). **(C)** The formation of NETs (white box) produced by Neu in LPS-treated mouse was observed under a two-photon confocal microscope (APC-750-LY6G, red and nuclear-sytox green, green) *in vivo*. Quantification of NETs formation in the plasma was assessed by dsDNA **(D)** and neutrophil elastase coupled dsDNA **(E)**. Error bars represent mean ± S.D. n = 3, ***p < 0.001,****p < 0.0001 by Two-way ANOVA. Each experiment was repeated three times.

We tested the production of NETs by human peripheral blood Neu when co-cultured with mucosal epithelial cells lines NCM460. Neu produced fuller NETs when stimulated by LPS/ATP (**Figure 5A**, white arrow, **Supplementary Video 1**). At the same time, we showed that 2D cultured cells adhered and grew on a flat surface, while 3D cultured cells contact with other cells in three-dimensional space (**Supplementary Figure 4A**, white arrow, **Supplementary Video 2**).

When co-cultured with C2C12, mouse Neu produced NETs when stimulated with PMA as usual. However, more NETs seemed to be produced (**Figure 5B**, white arrow), and a lot of blebbing (**Figure 5B**, white arrowhead) was observed on C2C12 cells, but not in the negative control group. As shown in the **Supplementary Figure 4B** and **Supplementary Video 3**, we could observe that in the 3D culture system Neu and co-cultured C2C12 stretched deeper, but limited to the culture surface in the 2D culture system.

We also observed the production of a large number of NETs (**Figure 5C**, white box) in live mice under two-photon fluorescence microscopy. It can be observed that the production of NETs labeled with LY6G and sytox green in mouse liver increased after 3h of LPS injection in the tail vein. And dsDNA and NE-coupled dsDNA also increased accordingly (**Figures 5D, E**).

## Discussion

Neu have a short life span and are prone to apoptosis with characteristic morphological changes of nuclear condensation and cell shrinkage *in vitro* (34, 35). So it is great challenging to study the detailed molecular biological role of Neu due to their short life span and the inability to cryopreserve or expand them *in vitro* (36). Prolonging the lifespan of Neu and keeping them active is very important for the outbreak of inflammation. Moreover, the standard 2D cell culturing does not represent the actual microenvironment where cells reside in tissues. Especially during inflammation, the Neu infiltrate tissues where they perform their effector functions. However, the tissues are three-dimensional (3D) and thus performance of the cells might also be different than that in the 2D conditions (12). In the current study, mainly using the hydrogel, we estalished a 3D culture system for human and mouse peripheral blood Neu. We demonstrated that the 3D culture system was more suitable for Neu. The NETs phenomenon of Neu induced by PMA, and other DAMPs in the 3D culture system is similar to that *in vivo*, and the the 3D culture system could maintain better properties of Neu than the classcial 2D culture system.

Although the 3D culture of neutrophil-like cell line or primary Neu co-culture with other cells have been used for improving the viability of Neu (15, 16), however, the detailed phenomena of Neu in 3D culturing system have never evaluated. The hydrogel is a biodegradable and biocompatible porous material with the ability to maintain high water content, used in a wide range of medical applications (15, 37, 38), including the drug release carriers, corneal contact lenses, in bone and soft tissue regeneration, in reconstruction and in the treatment of burns (39). They have good permeability, allowing a variety of nutrients, gases, and metabolites to pass through them freely and resembling the extracellular environment of the body’s tissues (17). So It might be suitable for oberving the exact phenomena of Neu.

In current study, we characterized the morphology and activity of the human and mouse Neu cultured in the 3D culutring system based on hydrogel. Obviously, Neu showed better activity in 3D culture which was consistent with others’ study of other cells (18–21, 40). The human-derived Neu are the most frequent nucleated cells in the circulation (50-70% Neu), but the mouse-derived Neu are significantly less frequent in the blood (10-25% Neu) and exhibit functional differences, for mouse-derived Neu are resistant to intravenous immunoglobulin (IVIG)-mediated cell death (41). The two origins of Neu were used to evalute their activities and function in the 2D and 3D culture system, and the results demonstrated that the human-derived peripheral blood Neu were more active than mice under the same culture time and culture system.

According to the existing study, the apoptotic Neu displayed a loss of background functions: ability to spread and change shape, random migration and chemotaxis (41). As the cultivation time increases, Neu gradually undergo apoptosis after 24 hours, but the 3D culture system slowed down this process. It is well known that apoptotic Neu display profound loss of capacity to generate and release histotoxic products under external stimulation (42) ROS production is one of the mechanisms by which Neu kill microorganisms. In our results, the amount of ROS produced by Neu in 2D and 3D was lower than that in the first day, may be because the two groups had different degrees of apoptosis. As shown in **Figure 2D**, Neu had apoptosis after the first day with characteristic morphological changes of nuclear condensation and cell shrinkage.

NETs are web-like chromatin based structures that are released into the extracellular environment to aid in pathogen clearance, but they have also been implicated in excessive inflammation with resultant tissue damage, potentiation of autoimmunity, and promotion of vascular thrombosis (43). NETs were released when the Neu were simulated with PMA, LPS (28) or other DAMPs (44). In current study, our results showed the 3D systems could indeed assist Neu to maintain the function of NETs induced by LPS/ATP, IL-1β, IFN-γ, PMA and TNFα, respectilvey. We compared the differences of Neu between the 2D and 3D culture systems stimulated by IL-1β, TNFα, and LPS/ATP separately. Similar to the results of PMA, Neu stimulated by LPS/ATP, IL-1β and TNFα displayed more NETs in 3D culture and this suggests that our 3D culture of Neu may reduce the gap between *in vitro* an *in vivo* experimentations.

pt?>The 3D culture system could better simulated the interaction between cells and cells, cells and matrix *in vivo*, which may disappear in 2D culture, and provide more accurate depiction of cell polarization since in 2D the cells can only be partially polarized (11). When co-cultured with the colon mucosal epithelial cell line NCM460, Neu showed full NETs, and NCM460 showed membrane damage in 3D culture, while not in 2D culture. In the 3D culture movie, we can observed that the contact area between NCM460 and Neu was spatially multiplied in the 3D culture, while adjacent cells in 2D culture. The results were also confirmed in the co-culture of Neu and skeletal muscle cell C2C12.

In summary, the current study developed a better 3D culture system for Neu, and the 3D culture of Neu provides an opportunity to mimic the *in vivo* experiments.

## Data Availability Statement

The original contributions presented in the study are included in the article/**Supplementary Material**. Further inquiries can be directed to the corresponding authors.

## Ethics Statement

Ethical review and approval was not required for the study on human participants in accordance with the local legislation and institutional requirements. The patients/participants provided their written informed consent to participate in this study. The animal study was reviewed and approved by Animal Care and Use Committee of the Southern Medical University.

## Author Contributions

RL performed most of the experiments and contributed to planning the work, to the interpretation of data, and to writing the paper. ZW, JH, SH, YP, and YW contributed to the interpretation of data. QM supervised the study. All authors contributed to the article and approved the submitted version.

## Funding

This work was supported by the following funding sources: the National Natural Science Foundation of China (81772133, 82072100, and 81902444), the Guangdong Natural Science Fund (2020A1515011367 and 2020A1515010269), the Guangzhou Citizen Health Science and Technology Research Project (201803010034 and 201903010072).

## Conflict of Interest

YW was employed by Guangzhou Darui Biotechnology Co., Ltd.

The remaining authors declare that the research was conducted in the absence of any commercial or financial relationships that could be construed as a potential conflict of interest.

## Publisher’s Note

All claims expressed in this article are solely those of the authors and do not necessarily represent those of their affiliated organizations, or those of the publisher, the editors and the reviewers. Any product that may be evaluated in this article, or claim that may be made by its manufacturer, is not guaranteed or endorsed by the publisher.

## Supplementary Material

Supplementary Figure 1
**(A)** The formation of NETs when stimulated by PMA for 4 h in 2D and 3D culture systems which marked by NE (Alexa Fluor 488 dye, green) and nuclear (DAPI, blue) were observed in mouse peripheral blood Neu under a fluorescence microscope *in vitro*. **(B)** Mouse peripheral blood Neu released NETs marked by LY6G (PE, red) and nuclear (SYTO 13, green) when stimulated by LPS (2.5 μg/mL)/ATP (3 mM), IL-1β (20 ng/mL) and TNFα (20 ng/mL) for 4 h in 2D and 3D culture systems under a confocal microscope *in vitro*. **(C)** A significantly different dsDNA release was detected using fluorescent-based Picogreen assay. Error bars represent mean ± S.D. n = 3, *****p *< 0.0001 by Two-way ANOVA.

Supplementary Figure 2 Mouse peripheral blood Neu which were dyed by SYTO 13 (stain DNA in both live and dead eukaryotic cells) and PI (stain DNA in dead eukaryotic cells) during 6 days in 2D and 3D culture systems with or without the inhibition of apoptosis z-vad-fmk.

Supplementary Figure 3 Human peripheral blood Neu which were dyed by SYTO 13 and PI during 6 days in 2D and 3D culture systems with or without the inhibition of apoptosis z-vad-fmk.

Supplementary Figure 4 Immunofluorescence analysis using confocal laser microscopy of human peripheral blood Neu co-culture with NCM460 in 2D and 3D systems. **(A)** The location (white arrow) of cells in XZ and YZ view of the cell model were observed. Z-stacks were acquired using confocal fluorescence microscopy and were reconstructed using Olympus FV31S. **(B)** Three-dimensional reconstruction of C2C12 and Neu co-culture, and the cross-section of monolayer cells (white lines).

Supplementary Video 1 Three-dimensional image of human peripheral blood Neu co-culture with NCM460 in 2D and 3D culture systems. Neu produced NETs under LPS (2.5 μg/mL)/ATP (3 mM) for 4 h stimulation.

Supplementary Video 2 Three-dimensional image of human peripheral blood Neu co-culture with NCM460 in 2D and 3D culture systems and the location of cells in Z-stacks.

Supplementary Video 3 Three-dimensional image of Neu and C2C12 co-culture in 2D and 3D culture systems. Neu produced NETs (white arrows) under 20 μM PMA for 4 h stimulation, and the depth of cells spanned during culture (white lines).
