# Supplementary figures and images for: Neutrophils Culture in Collagen Gel System
Source: Front Immunol. 2022 Jan 24;13:816037. doi: 10.3389/fimmu.2022.816037 (PMC8818728; doi:10.3389/fimmu.2022.816037)

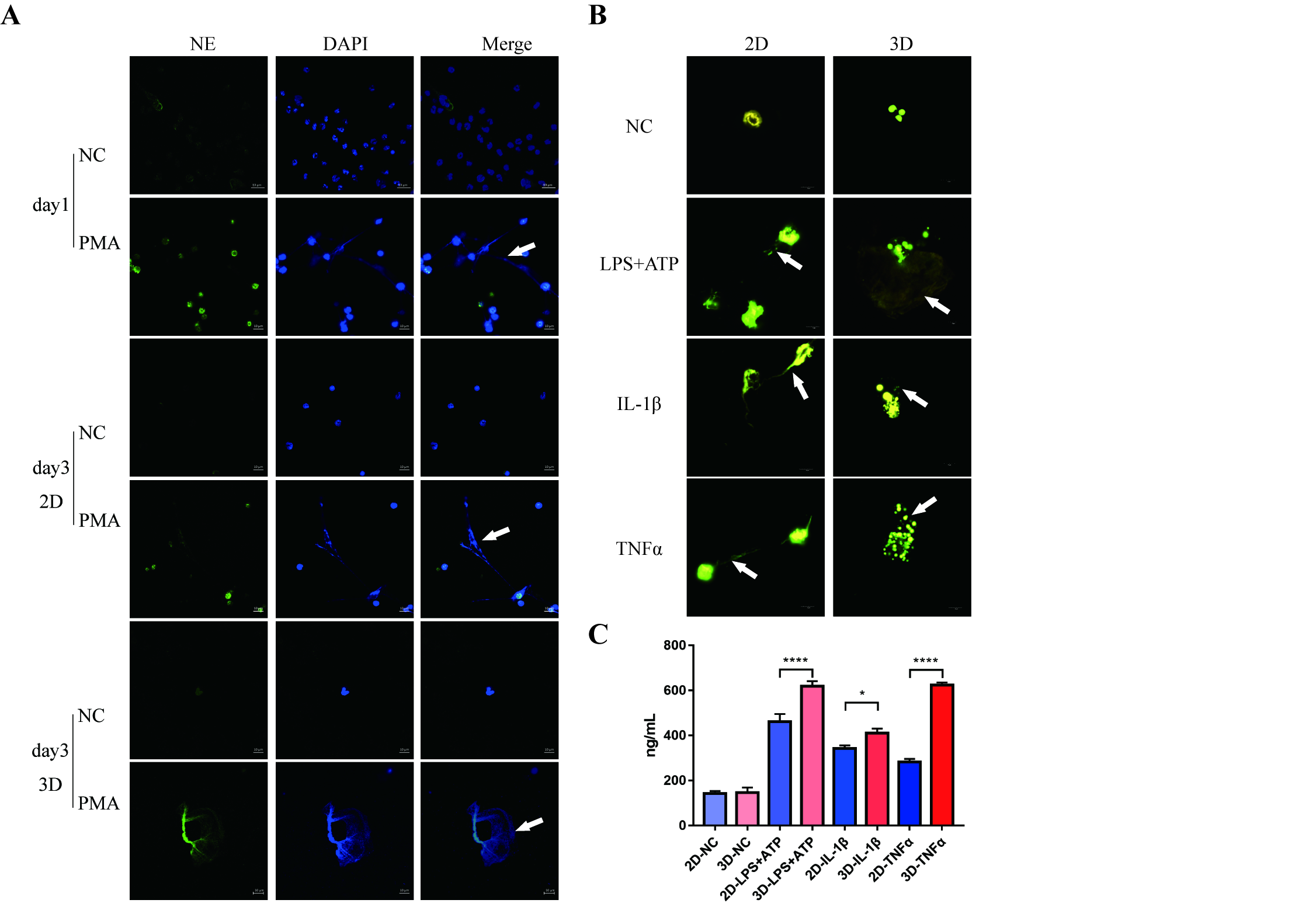

Supplement: Supplementary Figure 1 — (A) The formation of NETs when stimulated by PMA for 4 h in 2D and 3D culture systems which marked by NE (Alexa Fluor 488 dye, green) and nuclear (DAPI, blue) were observed in mouse peripheral blood Neu under a fluorescence microscope in vitro. (B) Mouse peripheral blood Neu released NETs marked by LY6G (PE, red) and nuclear (SYTO 13, green) when stimulated by LPS (2.5 μg/mL)/ATP (3 mM), IL-1β (20 ng/mL) and TNFα (20 ng/mL) for 4 h in 2D and 3D culture systems under a confocal microscope in vitro. (C) A significantly different dsDNA release was detected using fluorescent-based Picogreen assay. Error bars represent mean ± S.D. n = 3, ****p < 0.0001 by Two-way ANOVA. [file Image_1.tif]

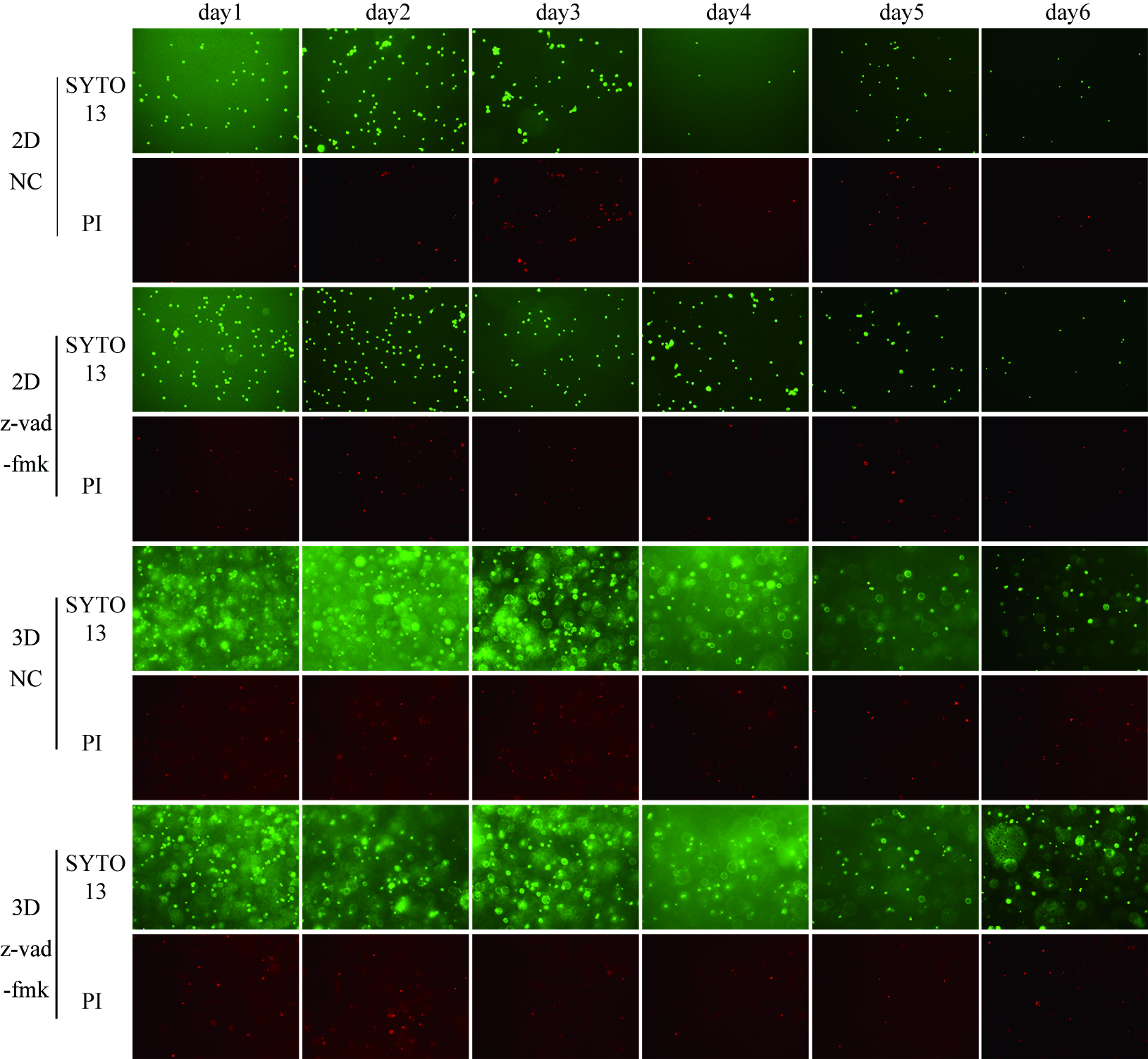

Supplement: Supplementary Figure 2 — Mouse peripheral blood Neu which were dyed by SYTO 13 (stain DNA in both live and dead eukaryotic cells) and PI (stain DNA in dead eukaryotic cells) during 6 days in 2D and 3D culture systems with or without the inhibition of apoptosis z-vad-fmk. [file Image_2.tif]

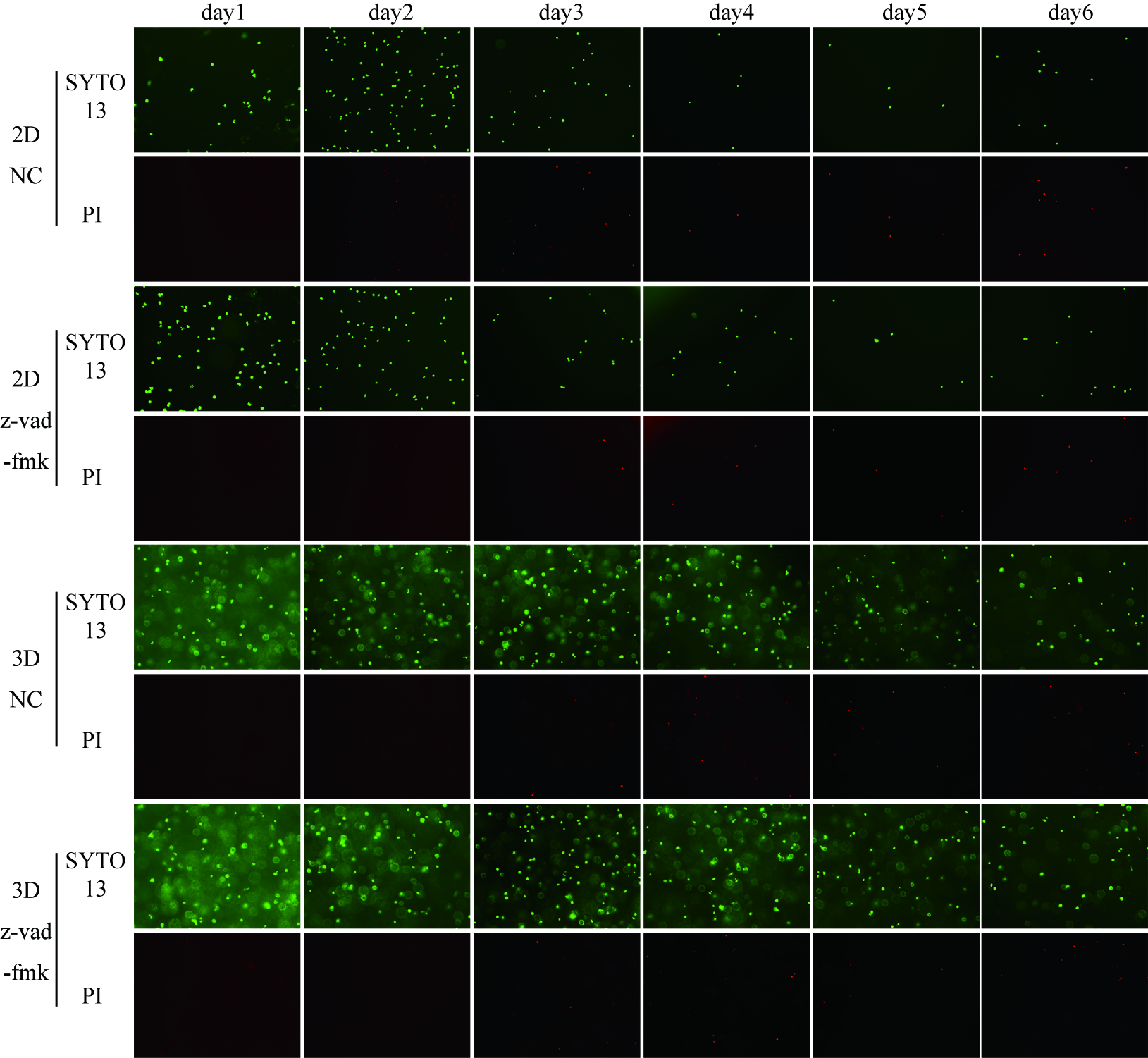

Supplement: Supplementary Figure 3 — Human peripheral blood Neu which were dyed by SYTO 13 and PI during 6 days in 2D and 3D culture systems with or without the inhibition of apoptosis z-vad-fmk. [file Image_3.tif]

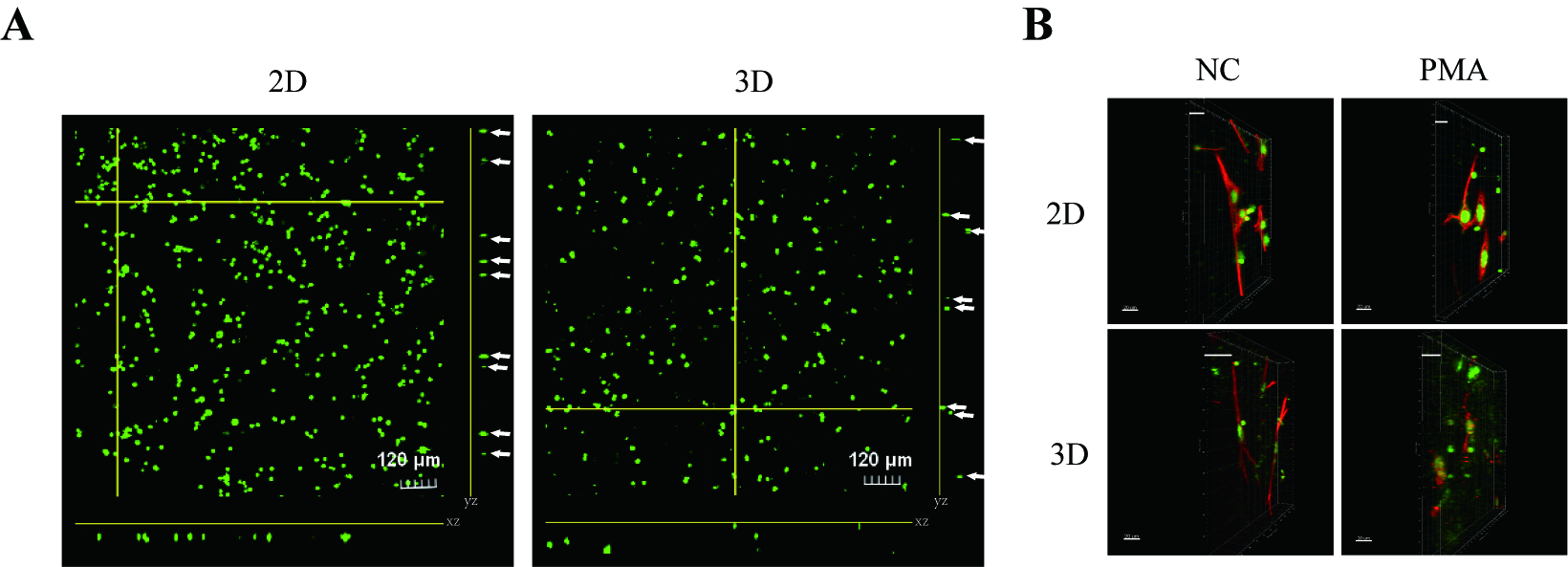

Supplement: Supplementary Figure 4 — Immunofluorescence analysis using confocal laser microscopy of human peripheral blood Neu co-culture with NCM460 in 2D and 3D systems. (A) The location (white arrow) of cells in XZ and YZ view of the cell model were observed. Z-stacks were acquired using confocal fluorescence microscopy and were reconstructed using Olympus FV31S. (B) Three-dimensional reconstruction of C2C12 and Neu co-culture, and the cross-section of monolayer cells (white lines). [file Image_4.tif]
